# Development of Microfluidic Culture System for Spatially Regulated Differentiation of Mesenchymal Stem Cells Toward Osteochondral Interface Regeneration

**DOI:** 10.3390/mi17091073

**Published:** 2026-09-10

**Authors:** Toshifumi Ohi, Shogo Miyata

**Affiliations:** 1 Graduate School of Science and Technology, Keio University, 3-14-1 Hiyoshi, Yokohama 223-8522, Japan; 2 Faculty of Science and Technology, Keio University, 3-14-1 Hiyoshi, Yokohama 223-8522, Japan

**Keywords:** microfluidic culture device, osteochondral interface, mesenchymal stem cell, concentration gradient

## Abstract

Regeneration of the osteochondral interface is essential for effective articular cartilage repair and the prevention of graft delamination. In this study, we developed a microfluidic culture device for the spatial regulation of mesenchymal stem cell (MSC) differentiation within a single-phase hydrogel by controlling concentration gradients of differentiation-inducing factors. The system comprised a PDMS culture chamber with dual-flow of culture media to generate concentration gradients of chondrogenic and osteogenic inductive factors. Numerical simulations were performed to characterize the flow velocity and distribution of factors aligned with the dual-flow direction in the hydrogel. The numerical results confirmed that the dual-flow successfully induced concentration differences between the opposite sides of the hydrogel. Bovine bone marrow-derived MSCs encapsulated in 3% agarose gel were cultured for 20 days under continuous dual-flow containing chondrogenic and osteogenic differentiation media. Histological analysis showed that the localized calcium deposition was aligned with the flow direction on the osteogenic differentiation side. In contrast, sulfated glycosaminoglycan (sGAG) synthesis was observed on the chondrogenic differentiation side. These results demonstrate that the developed culture system can successfully reconstruct an osteochondral-like interface from MSCs within a single-phase hydrogel.

## 1. Introduction

Articular cartilage is a highly specialized connective tissue that covers the articulating surfaces of bones. Its primary physiological functions are to provide a smooth, low-friction surface for joint movement and facilitate the transmission and dissipation of mechanical loads to the underlying bone [1,2,3,4]. From a structural viewpoint, this tissue is characterized by a dense extracellular matrix (ECM) with a sparse population of chondrocytes, which account for less than 10% of total tissue volume. The ECM consists of type II collagen fibers and aggrecan, a large proteoglycan that provides high hydration in cartilage tissue. Mature articular cartilage has a depth-dependent zonal organization that transitions from the superficial zone to the deep zone and culminates in a calcified cartilage layer as an osteochondral junction [5,6,7,8,9]. This calcified layer serves as a mechanical interface, anchoring the resilient cartilage to the stiff subchondral bone through the integration of collagen fibers into the mineralized matrix, thereby preventing tissue delamination under complex loading conditions.

Despite its remarkable mechanical durability, articular cartilage possesses an extremely limited intrinsic capacity for self-repair owing to its avascular nature [4,8,9,10]. Therefore, when the cartilage is damaged by trauma or degenerative diseases, the lesion fails to heal spontaneously, leading to osteoarthritis (OA). OA is a major joint disease characterized by the progressive degeneration of articular cartilage, breakdown of the collagen network, and depletion of proteoglycans. These pathological changes lead to severe joint pain and loss of mobility, significantly reducing the quality of life. Moreover, long-term therapeutic strategies are required to treat OA as the population ages.

Current clinical interventions for cartilage defects range from pharmacological treatments to surgery. Marrow stimulation techniques that generate microfractures in the subchondral bone are commonly used to recruit mesenchymal stem cells (MSCs) from the bone marrow for cartilage tissue regeneration [8,11]. However, this technique often results in the formation of fibrocartilage that lacks the mechanical properties of native hyaline cartilage. Total joint replacement (TJR) remains the “gold standard” for end-stage OA; however, artificial joints have a limited lifespan of approximately 15–20 years and involve highly invasive surgery.

Consequently, tissue engineering approaches have been developed as alternative therapies that involve culturing autologous chondrocytes in vitro to create a three-dimensional (3D) tissue designed to replace damaged tissue [12,13,14,15,16,17,18]. In clinical studies, chondrocyte sheets or cultures in atelocollagen gels have been transplanted to treat osteochondral defects [12,13,14,15]. However, they face persistent challenges, including donor site morbidity, limited cell availability, and, most importantly, the risk of graft delamination at the interface between the repaired cartilage and underlying bone [19]. To overcome these limitations, tissue engineering has shifted toward the fabrication of integrated osteochondral complexes that include both cartilage and bone layers, as well as the use of MSCs as a cell source. Reconstructing this complex interface is essential to ensure stable graft integration and prevent graft delamination.

Previous studies have reported various biomaterial strategies for developing a heterogeneous structure encompassing both articular cartilage and subchondral bone [9,20,21,22,23,24,25]. For example, bilayered and multilayered scaffolds designed for multi-lineage cell cultures have been fabricated by laminating electrospun polymeric microfibers onto particulate-templated porous polycaprolactone (PCL) matrices [24]. Furthermore, functionally graded pore structures have been widely investigated; bilayered constructs featuring smaller pore sizes (100–200 μm) in the chondrogenic zone and larger pore sizes (300–450 μm) in the osteogenic zone have demonstrated promising therapeutic efficacy in repairing osteochondral defects in vivo [25]. These gradient constructs exhibit superior mechanical stiffness and enhance the localized osteogenic and chondrogenic differentiation of MSCs compared to conventional scaffolds with uniform porosity. However, a major limitation of these advanced manufacturing methods is their reliance on highly complex multistep fabrication procedures, precise multi-material alignment, and extended in vitro pre-culture durations before implantation.

This study aims to develop a novel microfluidic culture device capable of rapidly and efficiently reconstructing the osteochondral interface within a single-phase hydrogel containing MSCs. We hypothesize that active flow control would establish stable concentration gradients of chondrogenic and osteogenic differentiation factors, enabling spatial regulation of MSC differentiation within a single-phase material. Our system utilizes a polydimethylsiloxane (PDMS)-based culture chamber integrated with a syringe pump to maintain a continuous, low-velocity, dual-flow differentiation medium. This approach simulates the natural biochemical gradient of the osteochondral junction while simplifying fabrication and eliminating the delamination risks associated with multi-material scaffolds. This study also evaluates the performance of the culture device through numerical simulations to predict the distribution of inducing factors. It validates the results through histological and biochemical assays of MSC-laden agarose gels cultured under continuous dual-flow conditions.

## 2. Materials and Methods

### 2.1. Device Design and Fabrication of Dual-Flow Culture System

**Figure 1:**
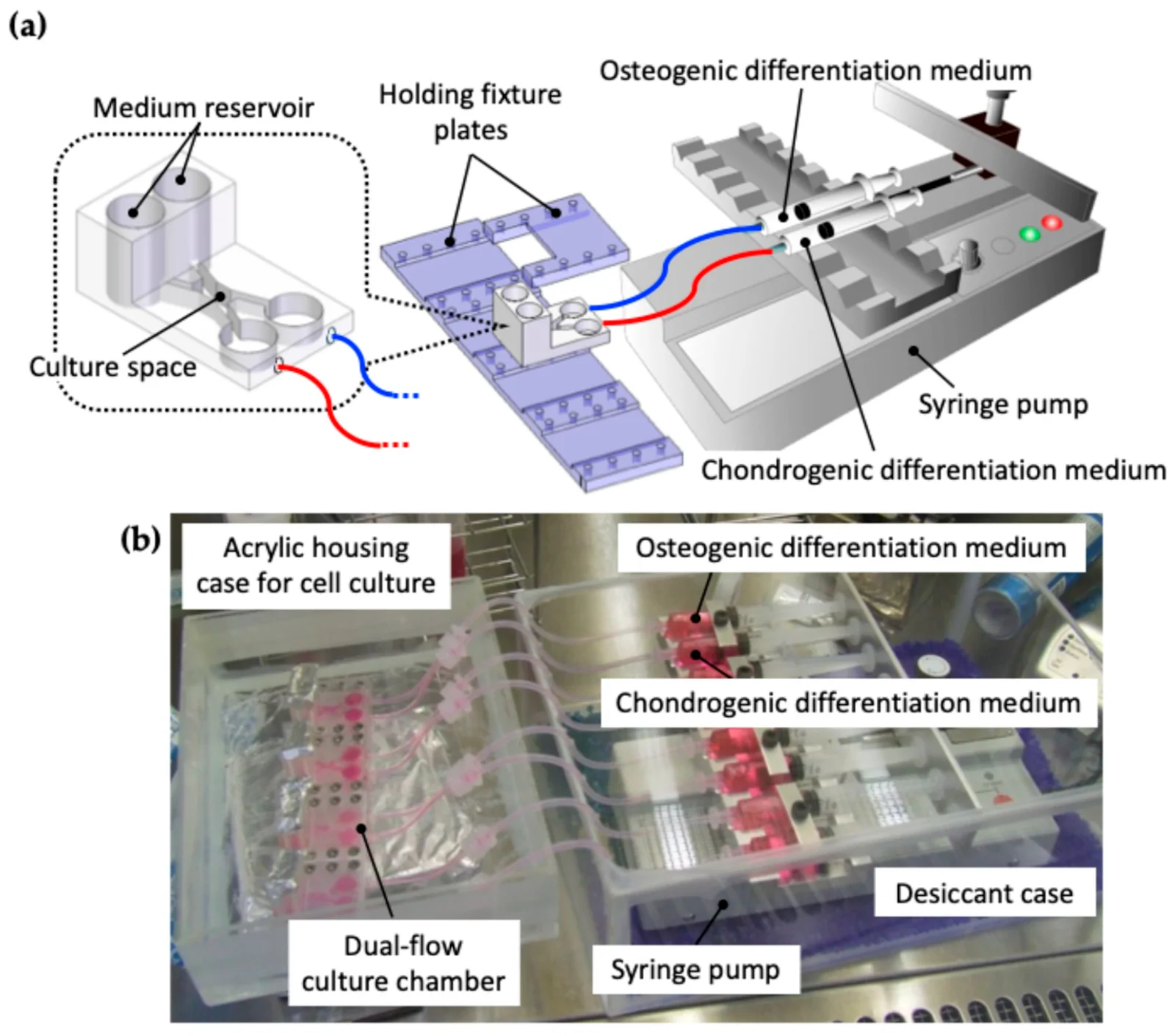
Overview of the microfluidic device and culture setup. (**a**) Schematic of a single culture chamber with a central cell-culturing space connected to dual fluidic channels to form parallel flows. (**b**) Photograph of the multiplexed culture system comprising four parallel chambers connected to a single syringe pump.

In this study, the microfluidic culture system is designed to establish stable concentration gradients of two differentiation media within a single-phase hydrogel by generating two parallel fluid flows adjacent to the cell-laden hydrogel. Therefore, the localized biochemical microenvironment for directing distinct cell differentiations is controlled. The system primarily consists of a custom-made PDMS culture chamber and a syringe pump (AS ONE, Tokyo, Japan). The PDMS culture chamber features a central cell-culturing space connected to two independent fluidic channels, equipped with two sets of inlets and outlets to introduce two distinct differentiation culture media (Figure 1a).

The chamber was fabricated using a molding technique. Briefly, a PDMS prepolymer solution (SYLGARD 184, Dow Corning Toray, Tokyo, Japan) was thoroughly mixed with its curing agent at a weight ratio of 10:1, degassed under vacuum, and poured into a custom-made mold. The mixture was then cured at 60 °C for 2 h to form the elastomeric microfluidic chamber. The resulting PDMS chamber featured a dual-flow structure with two sets of independent inlets and outlets. The central culture space, where the cell-laden gel was later placed, had a length (L) of 6.0 mm, a width (w) of 2.3 mm, and a height of 5 mm.

To precisely regulate flow rates, each inlet was connected to a syringe pump via silicone tubing, and each outlet was connected to a waste reservoir to collect the effluent media. For the experimental operation, the PDMS component was mechanically sandwiched between rigid polycarbonate-holding fixtures to ensure a leak-proof seal and structural stability during long-term perfusion culture. To facilitate the statistical analysis, four culture chambers were multiplexed into an array configuration (Figure 1b), connected in parallel to a syringe pump, and placed entirely within a standard humidified CO_2_ incubator (37 °C, 5% CO_2_) for the duration of the culture period.

### 2.2. Numerical Analysis for Concentration Gradient of Differentiation Factors in Hydrogel

**Figure 2:**
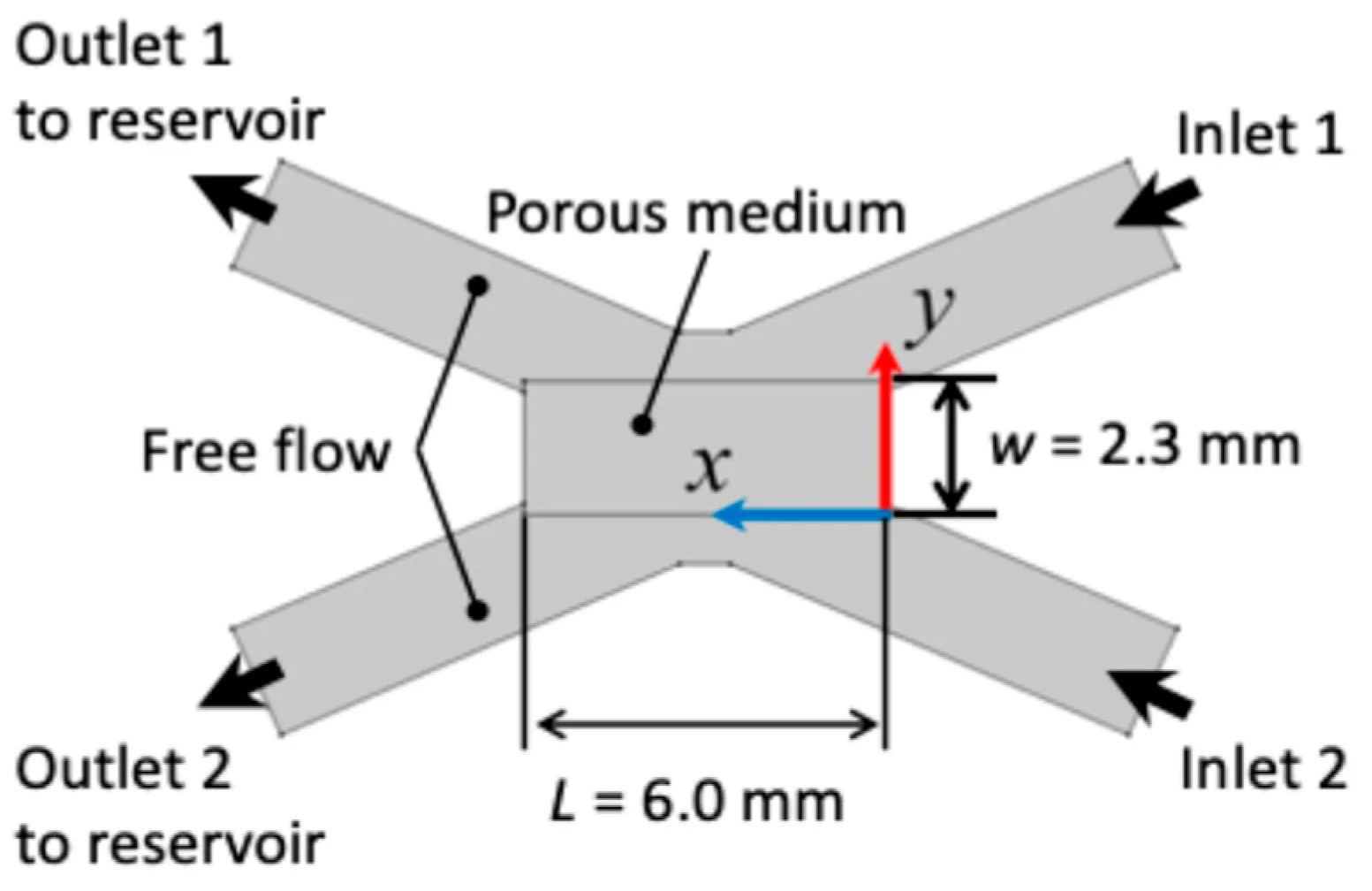
Two-dimensional simulation domain configuration. The model illustrates the horizontal center plane of the hydrogel, distinguishing the free-flow region (liquid area) and the porous flow region (porous area).

To analyze the formation of concentration gradients within the hydrogel, a numerical simulation was performed using COMSOL Multiphysics v4.2a (COMSOL Inc., Burlington, MA, USA). A two-dimensional model representing the horizontal center plane of the gel was established to evaluate the distribution of inductive factors under continuous-flow and static culture conditions. The model consisted of two domains: the free-flow region (liquid area), where the culture medium was supplied, and the porous flow region (porous area) representing the agarose gel (Figure 2). The physical properties and numerical parameters used in the simulation are summarized in Table 1.

For the free-flow region, the following steady-state incompressible Navier–Stokes equations were applied:

(1) $$\begin{array}{l} \rho_{L}\left(u\cdot \nabla \right)u=-\nabla p+\mu_{L}\nabla^{2}u,\\ \nabla \cdot u=0, \end{array}$$

where *ρ*_L_ denotes the density of the fluid (1.0 × 10^3^ kg/m^3^), ***u*** is the velocity vector, *p* is the pressure, and *μ*_L_ is the dynamic viscosity (1.0 × 10^−3^ Pa·s). For the porous flow region (agarose gel; length L = 6.0 mm, width w = 2.3 mm), the Brinkman equations with the Forchheimer correction term were utilized to account for the resistance within the porous structure:

(2) $$\frac{\mu_{G}}{\kappa }u=-\nabla p+\frac{\mu_{G}}{\varepsilon_{p}}\nabla^{2}u-\frac{\rho_{G}\varepsilon_{p}C_{f}}{\sqrt{\kappa }}u\left|u\right|,C_{f}=\frac{1.75}{\sqrt{150{\varepsilon_{p}}^{3}}},$$

where *ρ*_G_ is the density of agarose gel (porous media) (1.0 × 10^3^ kg/m^3^), *μ*_G_ is the dynamic viscosity (1.0 × 10^−3^ Pa·s), *κ* is the permeability (1.0 × 10^−16^ m^2^), and *ε*_p_ is the porosity (0.97) of the 3% agarose gel [26].

The spatial distribution of the differentiation-inducing factors was calculated using the following steady-state convection-diffusion equation:

(3) $$\left(u\cdot \nabla \right)uC=D\nabla^{2}C,$$

where *C* denotes the concentration of the inducing factor, and *D* is the diffusion coefficient, set at 1.0 × 10^−9^ m^2^/s in the fluid and 1.8 × 10^−10^ m^2^/s within the gel (porous media) [27].

The boundary conditions were set based on experimental parameters: the normal inflow velocity at the inlets was 1.67 × 10^−6^ m/s (corresponding to a flow rate of 1.0 μL/min), the pressure at the outlets was zero, and a no-slip condition was applied to the walls. The initial inlet concentrations (*C*_0_) based on the molecular weights and concentrations of differentiation-inducing factors were obtained as follows:

(4) $$C_{0}=\frac{\sum_{k=1}^{n}M_{k}C_{k}}{\sum_{k=1}^{n}M_{k}},$$

*n* denotes the total number of differentiation-inducing factors, and *M_k_* and *C_k_* represent the molecular weight and concentration of the *k*-th inducing factor, respectively. This formulation assumes that multiple differentiation-inducing factors are uniformly distributed within the medium and behave collectively as a single chemical species. From the concentrations of the main inducing factors in both the osteogenic and chondrogenic differentiation media (Table 2), the initial concentrations were determined to be 5.0 mM for the osteogenic side and 5.4 μM for the chondrogenic side inlets.

### 2.3. Isolation and Expansion of Bovine MSCs

Bovine bone marrow-derived MSCs (bMSCs) were isolated from the femurs of 4–6-week-old calves obtained from a local abattoir within 2 days of post-slaughter [28]. Under sterile conditions, the bone marrow was exposed, and 3–4 mL of marrow aspirate was collected using an 18-gauge needle attached to a 30 mL syringe. The aspirate was resuspended in phosphate-buffered saline (PBS) to a total volume of 15 mL and centrifuged at 2000 rpm for 7 min. The cell pellet was collected and washed twice with PBS to remove residual red blood cells.

The collected cells were seeded onto 100 mm culture dishes and expanded in a growth medium consisting of Dulbecco’s Modified Eagle Medium with low glucose (DMEM-LG) supplemented with 10% fetal bovine serum (FBS), 10 ng/mL basic fibroblast growth factor (bFGF), and 1% antimycotic-antibiotic solution. Cultures were maintained in a humidified incubator at 37 °C and 5% CO_2_. Non-adherent cells were removed by the first medium exchange 3 days after seeding, and the growth medium was replaced every 3–4 days. Upon reaching confluence (typically within 2–3 weeks), the adherent bMSCs were harvested using 0.25% trypsin-EDTA and passaged. Passages 2–4 were used to fabricate the bMSC-containing agarose gel layers.

### 2.4. Preparation of MSC-Laden Hydrogel Layer in PDMS Chamber and Perfusion Culture Conditions

To fabricate a 3D culture, expanded bMSCs were encapsulated in an agarose hydrogel. Briefly, a 6% (*w*/*v*) low-melting-point agarose solution (Type VII, Sigma-Aldrich, Burlington, MA, USA) was prepared in PBS and maintained at approximately 37 °C to prevent gelation. The bMSC suspension (1.5 mL) was mixed with an equal volume of a 6% agarose solution, resulting in a final concentration of 3% agarose with a cell density of 3.8 × 10^6^ cells/mL.

The gel layer within the PDMS chamber was fabricated using a precision molding technique to ensure open flow channels. Prior to the injection of the agarose solution, two 0.5 mm-thick ABS sheets were placed inside the culture chamber as separators to define the boundaries of the gel layer and flow channels. The MSC-laden agarose mixture was injected into the central space between separators and allowed to solidify for 5 min at room temperature. After gelation, the separators were carefully removed, and the PDMS chamber was sandwiched between polycarbonate-holding plates to establish a dual-flow perfusion system.

To induce spatially controlled differentiation, two types of differentiation media were prepared using a basal differentiation medium consisting of Dulbecco’s Modified Eagle Medium with high glucose (DMEM-HG) supplemented with 100 nM dexamethasone and 1% antimycotic-antibiotic solution. The osteogenic differentiation medium was prepared by adding 10% FBS, 10 mM β-glycerophosphate, and 0.05 mM L-ascorbic acid to the basal medium [29]. The chondrogenic differentiation medium was prepared by supplementing the basal medium with 1% ITS+ premix (6.25 μg/mL insulin, 6.25 μg/mL transferrin, 6.25 μg/mL selenious acid, 1.25 mg/mL bovine serum albumin, and 5.35 μg/mL linoleic acid), 0.2 mM L-ascorbic acid, 1.0 mM sodium pyruvate, and 10 ng/mL (0.4 nM) TGF-β3 [30].

**Figure 3:**
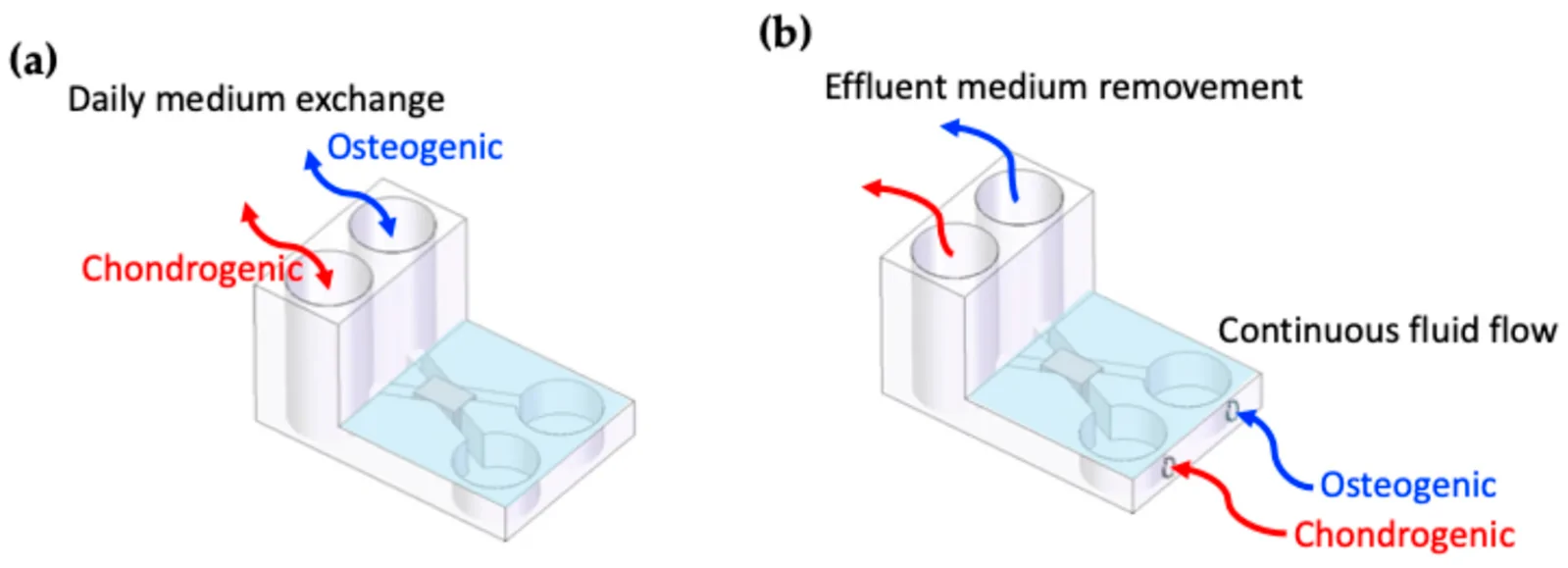
Schematic of fluidic operations for the two experimental groups. (**a**) Static (St) group configuration representing daily manual medium exchange. (**b**) Flow group configuration illustrating continuous media perfusion via a syringe pump. Arrows denote the fluid pathways for each culture condition.

The MSC-laden gel constructs were divided into two experimental groups: static (St) and Flow. In the St group, 1.5 mL of each differentiation medium was added to the respective culture reservoirs and replaced manually every day (Figure 3a). In the Flow group, the dual-flow perfusion was maintained by a syringe pump at a constant flow rate of 1.0 μL/min (Figure 3b). For the Flow group, the effluent media in the outlet reservoirs were removed daily, and the syringes were refilled every 2 days to ensure continuous delivery. To protect the mechanical components from high humidity, the syringe pump was housed in an acrylic case containing silica gel desiccants. All experiments were conducted in a humidified incubator at 37 °C and 5% CO_2_ for 20 days.

### 2.5. Histological and Biochemical Analyses

After 20 days of culture, the MSC-laden hydrogel constructs were harvested and subjected to histological and biochemical evaluations. For histological analysis, the specimens were fixed in 4% paraformaldehyde at 4 °C for 24 h and subsequently cryoprotected by sequential immersion in 10%, 20%, and 30% sucrose solutions for 24 h. The samples were then embedded in OCT compound (Sakura Finetek Japan, Tokyo, Japan), frozen at –80 °C, and sectioned into 100 μm thick slices using a cryomicrotome (HM525, Thermo Fisher Scientific, Waltham, MA, USA). Chondrogenic and osteogenic differentiation were evaluated by staining the sections with 0.05% toluidine blue (pH 2.5) to detect sGAG synthesis and 2% Alizarin Red S (pH 4.2) to detect calcium deposition. Images were captured using a microscope (CKX41, Olympus, Tokyo, Japan) equipped with a CCD camera (DP71, Olympus, Tokyo, Japan). To extract quantitative data from the microscopic images of stained sections, image processing software, ImageJ ver.1.54 (National Institutes of Health, Bethesda, MD, USA), was used. The images were binarized, and the stained area was obtained by applying a grayscale threshold to identify positively stained areas. A rectangular region of interest (ROI) was inscribed within the sample construct. The rectangular region was subsequently divided into four equal regions parallel to the flow direction. The stained area ratio was then calculated for each region as the percentage of the positively stained area relative to the total area of that corresponding region.

For biochemical characterization, the cultured constructs were bisected into the osteogenic and chondrogenic differentiation sides using a scalpel. After recording the wet weight, the samples were dried for 24 h using a freeze dryer (VA-140S, Taitec, Koshigaya, Japan) to determine the water content. The dried samples were pulverized using a freeze mill (SK mill, Tokken, Kashiwa, Japan) and solubilized in 1% Triton X-100 for 1 h to facilitate cell lysis. The total DNA content in the cultured gel was measured using a Hoechst 33258 fluorometric assay (excitation: 365 nm, emission: 458 nm; FP-750, JASCO, Tokyo, Japan), with cell numbers calculated based on a conversion factor of 7.7 pg DNA per cell [31]. Additionally, sulfated glycosaminoglycan (sGAG) production was quantified using a 1,9-dimethylmethylene blue (DMMB) assay at 540 nm (iMark, Bio-Rad, Hercules, CA, USA) [32,33]. In contrast, calcium content was determined using a calcium colorimetric assay kit (Biovision, Milpitas, CA, USA). All biochemical results were normalized to the cell number calculated from the DNA assay to evaluate the chondrogenic and osteogenic differentiation of the cells on each side of the chamber.

## 3. Results

### 3.1. Numerical Characterization of Concentration Gradients of Differentiation-Inductive Factors

**Figure 4:**
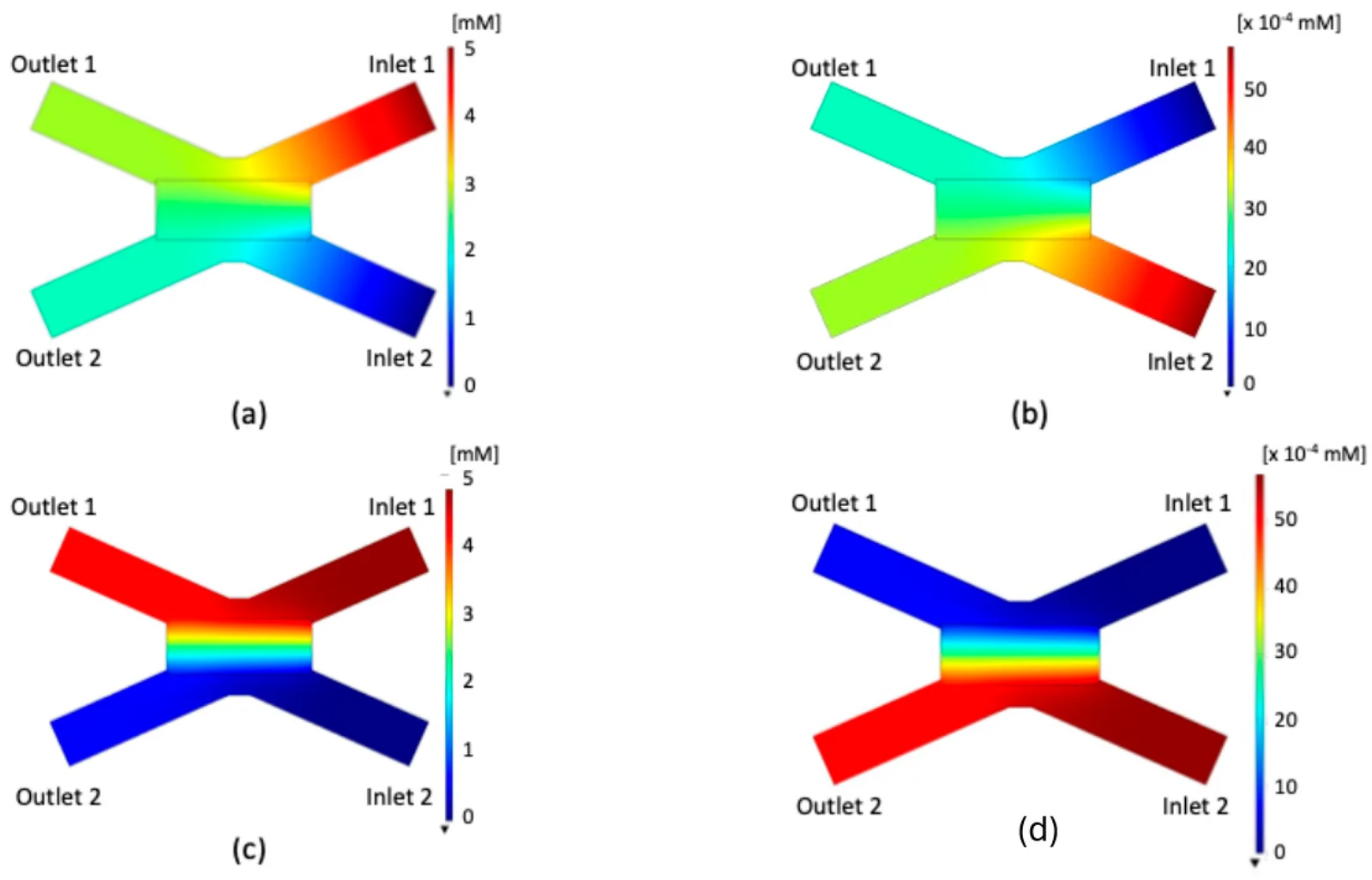
Numerical simulations of concentration gradient for differentiation-inducing factors within the hydrogel. Contour plots showing the spatial gradients of (**a**) osteogenic and (**b**) chondrogenic differentiation factors under St culture conditions. Corresponding contour plots under dual-flow perfusion (Flow) conditions for (**c**) osteogenic and (**d**) chondrogenic differentiation factors.

Numerical simulations were performed to evaluate the physiological environment of the hydrogels under dual-flow perfusion (Flow group) and static (St group) culture conditions. The velocity distribution analysis revealed that the flow velocity reached its peak at the intersections of the centerline (x = 3.0) and gel boundaries (*y* = 0 and *y* = w) because of a reduction in the flow channel width. The calculated Reynolds number was on the order of 10^−12^, confirming that the flow within the agarose gel remained laminar. The spatial distribution of the differentiation-inducing factors was significantly affected by the presence of continuous flow. In the St group, in which transport was dominated by diffusion, the concentration difference between the two sides of the gel decreased as the distance from the inlet increased (Figure 4a,b). In contrast, the Flow group maintained a stable concentration gradient across the entire length of the hydrogel, effectively preserving the initial concentration difference from the inlet to the outlet (Figure 4c,d).

### 3.2. Spatial Differentiation of bMSCs Cultured in Agarose Gel

**Figure 5:**
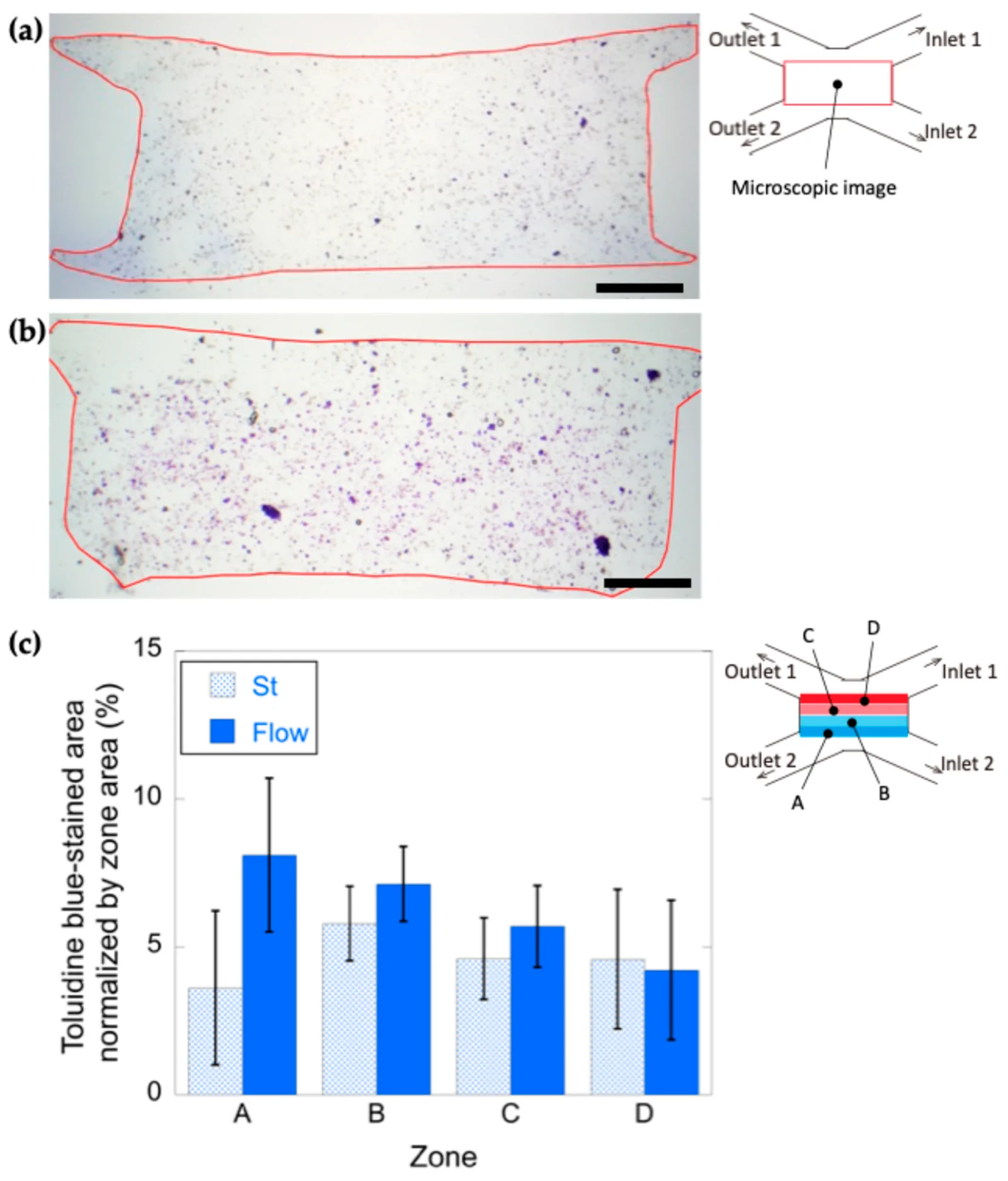
Histological evaluation of sulfated glycosaminoglycan (sGAG) production within the hydrogel constructs after 20 days of culture. Toluidine blue staining photographs of the (**a**) St group and (**b**) Flow group (red lines indicate construct boundaries). Scale bars: 1.0 mm. (**c**) Quantitative analysis of toluidine blue-positive area ratio in each region parallel to the flow direction (mean ± SD, *n* = 8).

Histological analysis was performed to evaluate the spatial distribution of chondrogenic and osteogenic matrix synthesis within the hydrogel constructs after 20 days of culture. Toluidine-blue staining, which was used to detect sGAG production, revealed that positive staining was primarily localized around individual cells and cell aggregates in both groups (Figure 5). In the St group, the cell aggregates were uniformly distributed and stained across both the chondrogenic and osteogenic sides, showing no spatial differences (Figure 5a). In contrast, the Flow group exhibited a distinct spatial distribution, with a substantially higher number of positively stained cell aggregates observed on the chondrogenic side than on the osteogenic side (Figure 5b). Quantitative analysis of the stained area ratio perpendicular to the flow direction further confirmed these spatial characteristics (Figure 5c). While the St group showed no notable change in the stained area ratio across the four areas, the Flow group tended to show a decrease in the ratio with increasing distance from the inlet of the chondrogenic differentiation medium.

**Figure 6:**
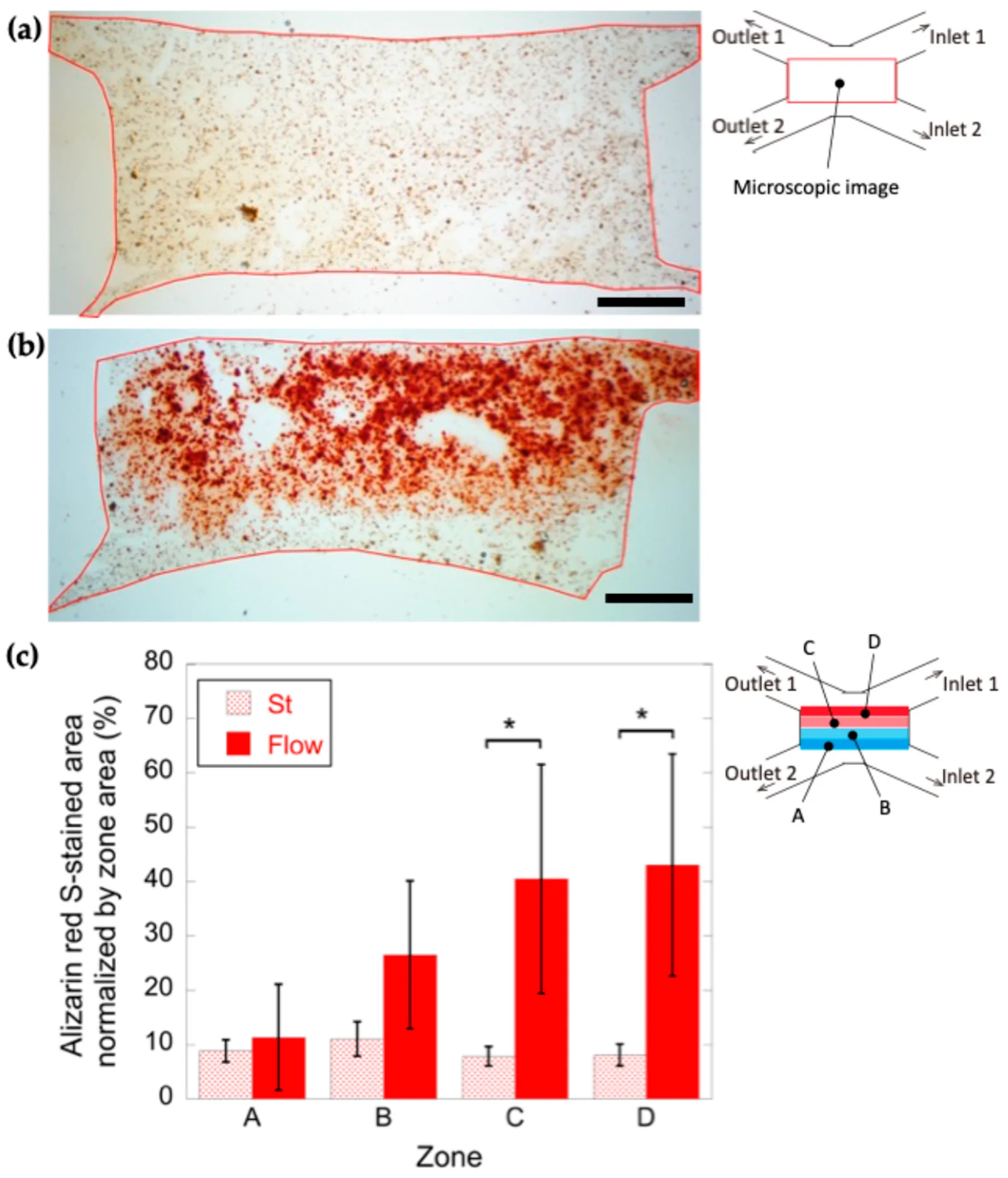
Histological evaluation and localization of calcium deposition within the hydrogel constructs after 20 days of culture. Alizarin Red S staining photographs of the (**a**) St group and (**b**) Flow group (red lines indicate construct boundaries). Scale bars: 1.0 mm. (**c**) Quantitative analysis of Alizarin Red S-positive area ratio in each region parallel to the flow direction (mean ± SD, *n* = 8). * indicates a significant difference (*p* < 0.05).

Alizarin Red S staining for calcium deposition revealed a marked difference between the experimental conditions (Figure 6). Small amounts of calcium were deposited in the St group with minimal variation across the gel (Figure 6a). In contrast, the Flow group exhibited intense red staining on the osteogenic side, particularly near the media inlet, where the concentration of osteogenic factors was the highest (Figure 6b). Quantitative analysis of the stained area ratio perpendicular to the flow direction further confirmed these spatial characteristics (Figure 6c). While the stained area ratio in the St group remained uniformly low across all four regions, that in the Flow group was significantly higher than the St group in the two proximal regions near the osteogenic medium inlet.

### 3.3. Biochemical Content of Cultured Constructs

**Figure 7:**
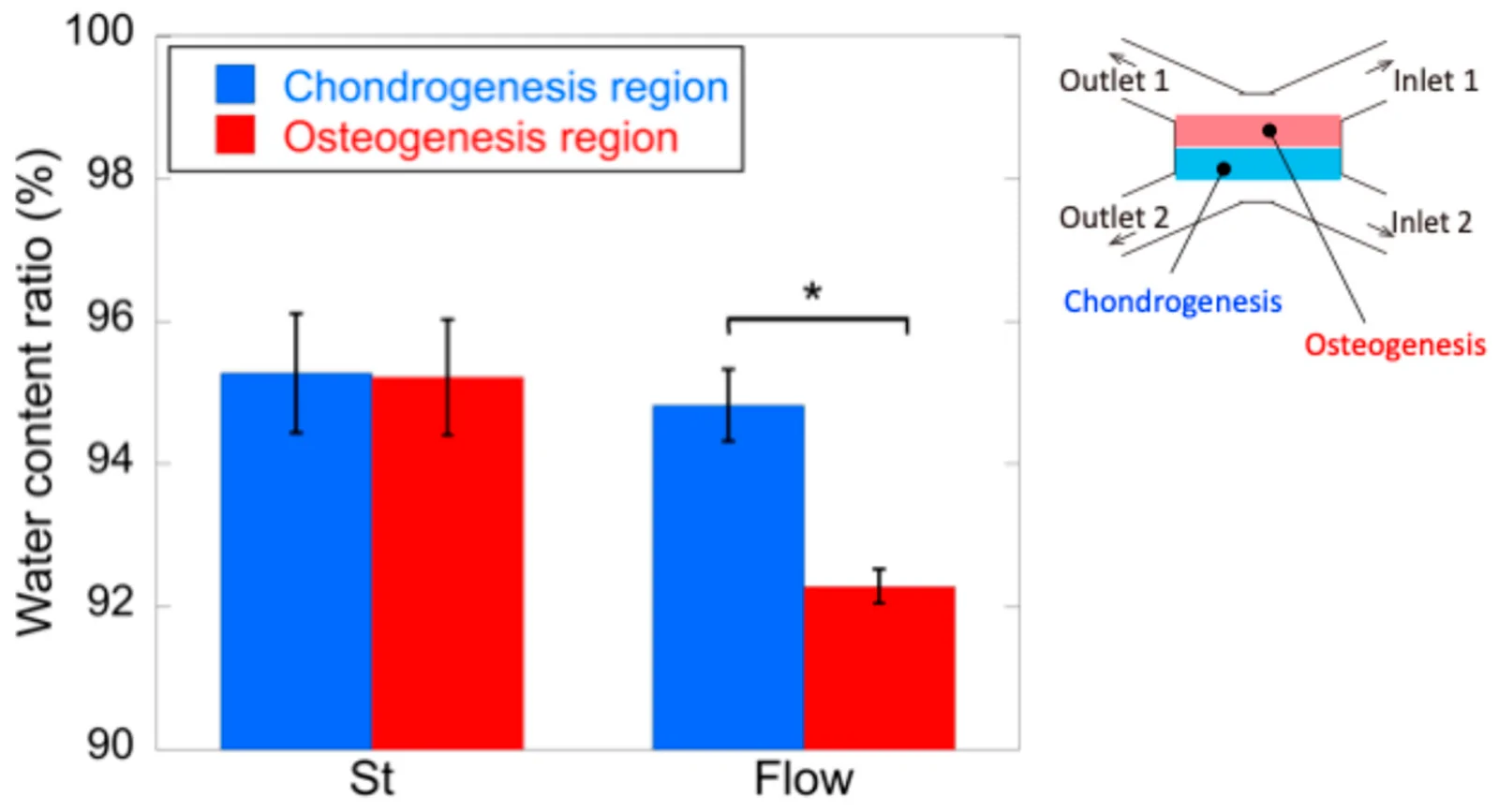
Quantitative evaluation of water content within hydrogel constructs after 20 days of culture. * indicates a significant difference (*p* < 0.05). Data are shown as mean ± SD, *n* = 8.

**Figure 8:**
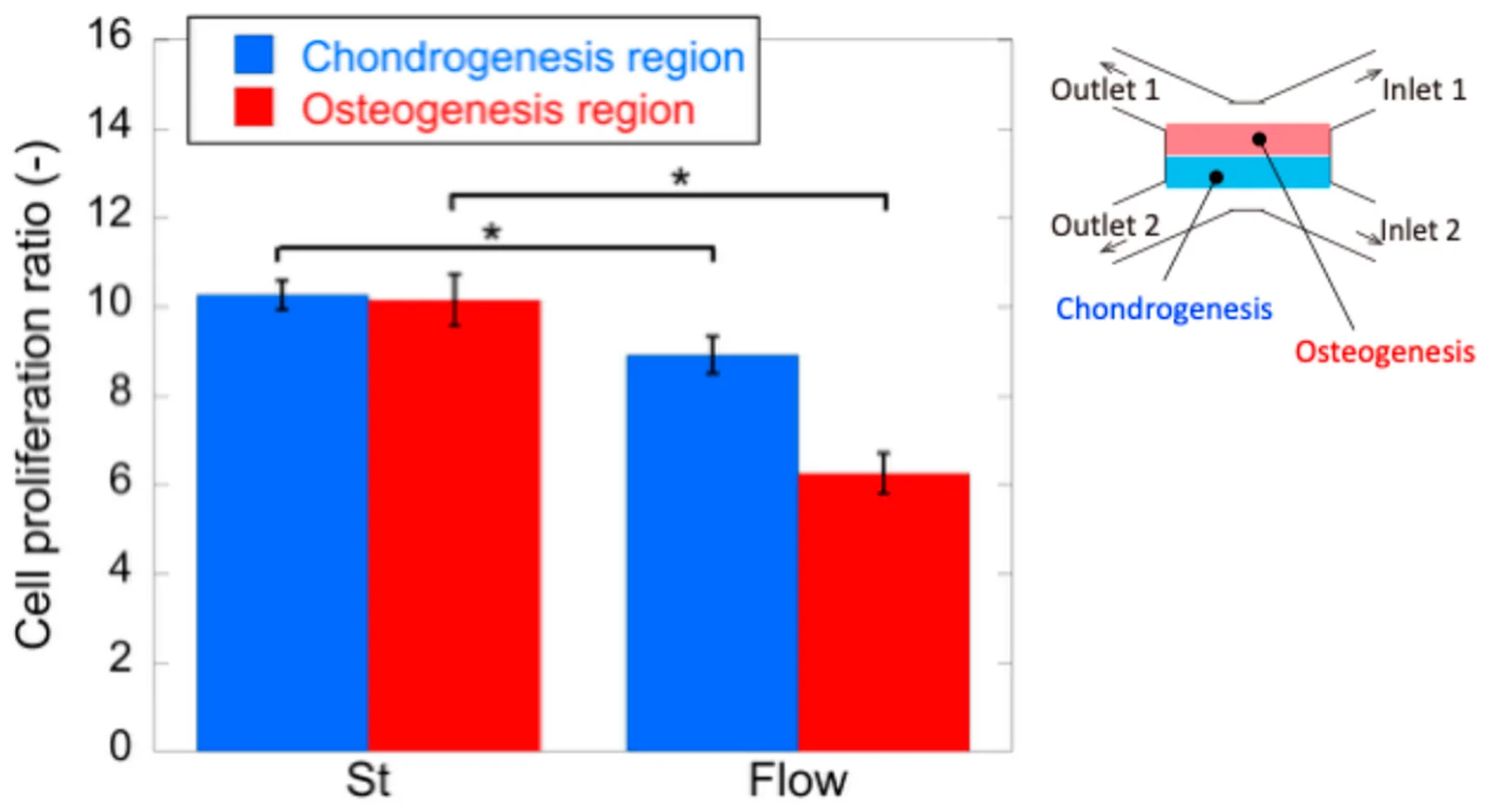
Quantitative evaluation of cell proliferation ratio determined by total DNA content after 20 days of culture. * indicates a significant difference (*p* < 0.05). Data are shown as mean ± SD, *n* = 8.

Biochemical analyses were performed after 20 days of culture to quantify cell proliferation and the production of tissue-specific extracellular matrix components. The water-content ratio after 20 days of culture was lower in the Flow group than in the St group. Within the Flow group, the osteogenic side exhibited lower water content than the chondrogenic side. In contrast, the St group maintained uniform water content across both sides (Figure 7). The cell proliferation ratio, determined by the total DNA content, showed that the Flow group exhibited significantly lower values on both the chondrogenic and osteogenic sides than the St group (*p* < 0.05) (Figure 8).

**Figure 9:**
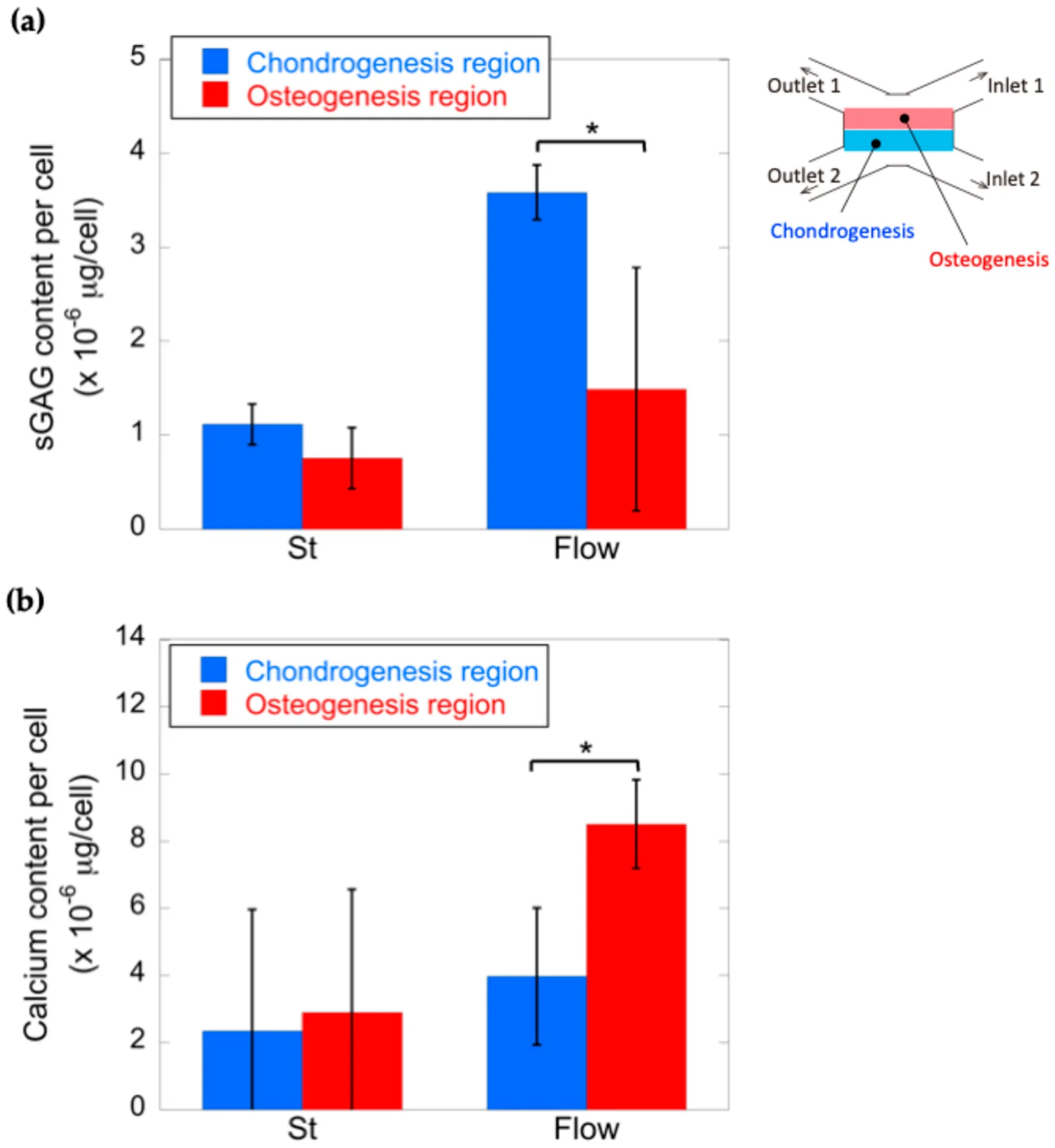
Quantitative evaluation of tissue-specific extracellular matrix components per cell after 20 days of culture. (**a**) sGAG production per cell and (**b**) calcium content per cell on the chondrogenic and osteogenic sides for the Static (St) and Flow groups. * indicates a significant difference (*p* < 0.05). Data are shown as mean ± SD, *n* = 8.

The production of sGAG per cell, a marker for chondrogenesis, reflects the spatial regulation of the culture environment. Sulfated-GAG production was significantly higher on the chondrogenic side than on the osteogenic side (*p* < 0.05) in both the St and Flow groups (Figure 9a). Calcium content per cell, a marker for osteogenesis, demonstrated a distinct spatial pattern in the Flow group. On the osteogenic side of the Flow group, calcium production was significantly higher than that on the chondrogenic side (*p* < 0.05), confirming a spatial gradient of mineralization. In contrast, the St group showed no significant difference in the calcium content between the two sides (Figure 9b).

## 4. Discussion

The numerical results demonstrated that the active perfusion system successfully established and maintained a concentration gradient of differentiation factors on each side of the single-phase hydrogel. While the concentration gradient in the St group dissipated toward the outlet because of the dominance of diffusion, the Flow group maintained a stable factor gradient. Therefore, the spatial organization of the reconstructed tissue in this device can be primarily attributed to the chemical concentration gradients of the differentiation factors.

Regarding the effect on cell proliferation, the Flow group exhibited significantly lower proliferation ratios compared to the St group. This suggests that the constant supply of induction factors and maintenance of stable gradients in the Flow group promoted advanced MSC differentiation and, coincidentally, suppressed cell proliferation [34]. In contrast, the relatively high proliferation observed in the St group may be attributed to the lack of sufficient biochemical cues to trigger a shift from a proliferative state to a differentiated phenotype. Furthermore, both microscopic and quantitative evaluations of sGAG and calcium depositions revealed distinct spatial patterns within constructs in the Flow group. The localization of these markers parallel to the perfusion direction demonstrates that MSC differentiation was successfully regulated in a spatially controlled manner. These spatial distributions qualitatively agreed with the steady-state concentration gradients estimated by the numerical analysis. While the discrete regional analysis across four regions limited quantitative comparison with the continuous numerical model, the observed biological responses reflected the predicted spatial transitions of differentiation factors.

Previous studies have established functional osteochondral interfaces predominantly by engineering gradients in scaffold composition, porosity, or mechanical stiffness to guide zone-specific cell differentiation [20,21]. In our perfusion-based system, the spatial gradient of calcium deposition was consistent with the zone-specific mineralization observed in these scaffold-based models. In contrast, although sGAG deposition in the Flow group exhibited a decreasing trend toward the osteogenic side, this gradient was less distinct than the cartilaginous matrix transitions reported in those studies. Characterization of water content provides further evidence of tissue-specific maturation. The Flow group exhibited a clear spatial difference in the water content, with the chondrogenic side maintaining a significantly higher value than the osteogenic side. Because native articular cartilage is characterized by high water content (approximately 80%) [6], the elevated water content on the chondrogenic side, supported by the water-retaining properties of sGAG, strongly corroborates the progression of chondrogenic differentiation in that region. These localized patterns of mineralization and sGAG deposition, combined with the corresponding water content profiles, reflect successful recapitulation of the transition structure from soft cartilage to a dense bone-like matrix.

Despite these promising results, several limitations of this study must be addressed. The two-dimensional numerical model utilized in this study provided a useful macroscopic view, but did not account for the 3D velocity profiles and wall effects near the top and bottom of the chamber. Future studies should employ 3D modeling to better predict spatial variations. Furthermore, while histological and biochemical assays provide strong evidence of differentiation, gene expression analysis, specifically evaluating chondrogenic markers such as Sox9 and Col2a1 (Type II collagen) [35], as well as the osteogenic marker Runx2 [36], is necessary to definitively characterize the phenotypic transition. Future research should focus on the mechanical characterization of the reconstructed interface, such as measuring the local Young’s modulus and shear strength. Such an evaluation is essential to quantitatively validate the mechanical integrity of the engineered junction and confirm that the spatial regulation of MSC differentiation has successfully reconstructed a structurally integrated transition zone to mimic the mechanical function of the native tidemark.

In summary, the microfluidic platform developed in this study successfully demonstrated simultaneous and spatially controlled differentiation of MSCs within a single-phase hydrogel, effectively recapitulating the transitional structure between soft cartilage and a dense bone-like matrix. By utilizing active perfusion to establish stable chemical concentration gradients, this system eliminates the distinct interfacial boundaries present in conventional multilayered scaffold strategies, thereby preventing structural delamination. Furthermore, this approach eliminates complex scaffold fabrication steps and provides a cost-effective, operationally simple methodology. In addition to the specific articular cartilage-to-bone interface engineered in this study, this microfluidic approach has significant potential for broader applications in musculoskeletal tissue engineering. By simply adjusting the combinations and flow rates of distinct biochemical factors, this platform can be readily adapted to construct other complex multi-tissue junctions, such as tendon-to-bone or muscle-to-bone interfaces, thereby offering a versatile and robust tool for advanced regenerative medicine.

## 5. Conclusions

In this study, we developed a microfluidic culture system capable of reconstructing an osteochondral interface within a single-phase agarose gel by spatially regulating MSC differentiation. Numerical simulations confirmed that active perfusion of the two distinct media successfully established and maintained stable concentration gradients of the differentiation factors across the hydrogel construct. Experimental validation after culturing under active perfusion demonstrated distinct spatial differentiation profiles, characterized by localized calcification on the osteogenic side. In contrast, chondrogenic matrix properties were effectively established on the opposite side. By eliminating distinct material boundaries, this single-phase platform avoids the critical risk of interfacial delamination inherent in traditional multilayered scaffolding techniques. These findings demonstrate that integrating active fluidic control with single-phase hydrogels provides a robust tool for studying interfacial biology and holds significant potential for engineering various structurally integrated multi-tissue junctions.

## Figures and Tables

**Table 1:** Parameters used for the concentration gradient analysis of differentiation factors in hydrogel.

Parameters	
Density of medium *ρ*_L_ (kg/m^3^)	1.0 × 10^3^
Medium dynamic viscosity *μ*_L_ (Pa·s)	1.0 × 10^−3^
Diffusion coefficient of medium *D* (m^2^/s)	1.0 × 10^−9^
Density of agarose gel *ρ*_G_ (kg/m^3^)	1.0 × 10^3^
Permeability of agarose gel *κ* (m^2^)	1.0 × 10^−16^
Porosity of agarose gel *ε*_p_ (-)	0.97
Diffusion coefficient of medium *D* (m^2^/s)	1.8 × 10^−10^

**Table 2:** Specifications of osteogenic and chondrogenic differentiation factors.

	Differentiation Factor	Concentration	Molecular Weight
Osteogenic differentiation	β-glycerophosphate	10 mM	172
	L-ascorbic acid	0.05 mM	176
Chondrogenic differentiation	TGF-β3	0.40 nM	25 × 10^3^
	L-ascorbic acid	0.20 mM	176
	sodium pyruvate	1.0 mM	110

## Data Availability

The datasets presented in this article are not readily available because they are part of an ongoing project. Access to the datasets should be directed to the corresponding author.

## Abbreviations

The following abbreviations are used in this manuscript:

bMSC	Bovine mesenchymal stem cell
ECM	Extracellular matrix
OA	Osteoarthritis
DMEM	Dulbecco’s Modified Eagle Medium
sGAG	sulfated glycosaminoglycan
